# Implementation and Comparison of Two Pharmacometric Tools for Model-Based Therapeutic Drug Monitoring and Precision Dosing of Daptomycin

**DOI:** 10.3390/pharmaceutics14010114

**Published:** 2022-01-04

**Authors:** Justine Heitzmann, Yann Thoma, Romain Bricca, Marie-Claude Gagnieu, Vincent Leclerc, Sandrine Roux, Anne Conrad, Tristan Ferry, Sylvain Goutelle

**Affiliations:** 1 Hospices Civils de Lyon, Groupement Hospitalier Nord, Service de Pharmacie, Hôpital Pierre Garraud, Service Pharmaceutique, 136 Rue du Commandant Charcot, 69005 Lyon, France; justine.heitzmann@chu-lyon.fr (J.H.); vincent.leclerc@aphp.fr (V.L.); 2 School of Management and Engineering Vaud (HEIG-VD), HES-SO University of Applied Sciences and Arts Western Switzerland, 1400 Yverdon-les-Bains, Switzerland; yann.thoma@heig-vd.ch; 3 Hôpital Nord-Ouest, Service de Médecine Interne et des Maladies Infectieuses, 69400 Villefranche sur Saône, France; RBricca@lhopitalnordouest.fr; 4 Hospices Civils de Lyon, Groupement Hospitalier Sud, Service de Biochimie et Biologie Moléculaire, UM Pharmacologie-Toxicologie, 69310 Pierre-Bénite, France; marie-claude.gagnieu@chu-lyon.fr; 5 Hospices Civils de Lyon, Groupement Hospitalier Nord, Hôpital de la Croix-Rousse, Service des Maladies Infectieuses et Tropicales, Centre Interrégional de Référence pour la Prise en Charge des Infections Ostéo-Articulaires Complexes (CRIOAc Lyon), 69004 Lyon, France; sandrine.roux01@chu-lyon.fr (S.R.); anne.conrad@chu-lyon.fr (A.C.); tristan.ferry@chu-lyon.fr (T.F.); 6 ISPB—Facultés de Médecine et de Pharmacie de Lyon, Université Lyon 1, University of Lyon, 69008 Lyon, France; 7 CIRI—Centre International de Recherche en Infectiologie, Inserm, U1111, Université Claude Bernard Lyon 1, CNRS, UMR5308, Ecole Normale Supérieure de Lyon, University of Lyon, 69007 Lyon, France; 8 Laboratoire de Biométrie et Biologie Evolutive, UMR CNRS 5558, Université Lyon 1, University of Lyon, 69100 Villeurbanne, France

**Keywords:** daptomycin, pharmacokinetics, therapeutic drug monitoring, model-informed precision dosing, bone and joint infection

## Abstract

Daptomycin is a candidate for therapeutic drug monitoring (TDM). The objectives of this work were to implement and compare two pharmacometric tools for daptomycin TDM and precision dosing. A nonparametric population PK model developed from patients with bone and joint infection was implemented into the BestDose software. A published parametric model was imported into Tucuxi. We compared the performance of the two models in a validation dataset based on mean error (ME) and mean absolute percent error (MAPE) of individual predictions, estimated exposure and predicted doses necessary to achieve daptomycin efficacy and safety PK/PD targets. The BestDose model described the data very well in the learning dataset. In the validation dataset (94 patients, 264 concentrations), 21.3% of patients were underexposed (AUC_24h_ < 666 mg.h/L) and 31.9% of patients were overexposed (C_min_ > 24.3 mg/L) on the first TDM occasion. The BestDose model performed slightly better than the model in Tucuxi (ME = −0.13 ± 5.16 vs. −1.90 ± 6.99 mg/L, *p* < 0.001), but overall results were in agreement between the two models. A significant proportion of patients exhibited underexposure or overexposure to daptomycin after the initial dosage, which supports TDM. The two models may be useful for model-informed precision dosing.

## 1. Introduction

Daptomycin is a cyclic lipopeptide antibiotic with bactericidal activity on Gram-positive bacteria discovered in 1980 [1,2]. The Food and Drug Administration (FDA) approved its use in 2003 for treating complicated skin and skin structure infections and *Staphylococcus aureus* bloodstream infections, including those with right-sided infective endocarditis. In those indications, the recommended dosing regimen ranges from 4 to 6 mg/kg [3]. Daptomycin is also used off-label in various indications, including bloodstream infections caused by Gram-positive agents others than *S. aureus* and bone and joint infections (BJI) [4]. Daptomycin use in patients with BJI is supported by its disposition in bone tissue. The average tissue to plasma concentration ratio was estimated at 54% and 14.1% in synovial fluid and bone, respectively [5]. In another study performed in 10 patients, the average ratio of area under the unbound concentration time-curve of daptomycin in bone over plasma was 1.08, which suggests that the bone exposure is similar to that of daptomycin unbound fraction in plasma [6]. The Infectious Diseases Society of America (IDSA) recognized daptomycin as an alternative to vancomycin for BJI, with a recommended dosage of 6 mg/kg [7]. Other studies have supported the use of daptomycin in patients with BJI, with higher dosages, up to 10 mg/kg [8,9,10].

However, increasing daptomycin doses raises concerns about safety [11,12,13]. It was shown that daptomycin muscular toxicity, as measured by elevation in serum creatinine phosphokinase (CPK), correlated with plasma trough concentration (C_min_). Bhavnani et al. suggested an upper bound for C_min_ target of 24.3 mg/mL [14].

On the other hand, the daptomycin antibacterial effect is also concentration-dependent. In a mouse model, the daptomycin killing effect correlated with both daily area under the concentration-time curve to the bacterial minimal inhibitory concentration (AUC_24h_/MIC) and maximal concentration over the MIC (C_max_/MIC) ratios [15]. For *Staphylococcus aureus,* an average AUC_24h_/MIC ratio of 666 mg/L was required to achieve a bactericidal effect. In a small clinical study, AUC_24h_/MIC < 666 was significantly associated with increased mortality in hospitalized patients [16]. Those established exposure–response and exposure–toxicity relationships are arguments supporting therapeutic drug monitoring (TDM) of daptomycin. TDM may be especially useful in patients with BJI who often receive high doses of daptomycin in order to control drug exposure. The use of PK models implemented into Bayesian dosing software is a relevant approach to analyze TDM results, estimate PK quantities such as the AUC, and compute individualized dosages for achieving PK/PD targets with maximum precision [17,18]. A number of programs are available, including BestDose and Tucuxi [19].

To our knowledge, no pharmacometric tool has been presented for dosage individualization of daptomycin. The objectives of this work were: (i) To implement and validate a nonparametric PK model of daptomycin for patients with BJI within the BestDose software; (ii) To compare the performance of this model to that of a published parametric model implemented in the Tucuxi software.

## 2. Material and Methods

### 2.1. Population Data

We report a retrospective analysis of data from patients who were treated by daptomycin for BJI between 2012 and 2016 and had TDM in our reference center for bone and joint infection in Lyon, France. We used two data sets: a learning set used to derive the nonparametric population model of daptomycin and a validation dataset. The learning dataset was described in a previous publication from our group, which focused on the influence of P-gp gene polymorphism on daptomycin PK (*n* = 81 patients) [20]. Unpublished data obtained in patients who did not undergo P-gp genotyping were used as the validation dataset (n = 94 patients). All patients were enrolled in a cohort study after information. This cohort study was subject to a declaration to the local Commission for Data Protection and Liberties (number 17-057) and is registered on ClinicalTrial.gov (number NCT03134521). In addition, patients’ written informed consent was obtained for P-gp genotyping, in accordance with French regulation.

### 2.2. Therapeutic Drug Monitoring of Daptomycin

Blood samples for daptomycin TDM were obtained during hospitalization or follow-up visit at the BJI center. In most patients, TDM was performed on several occasions to control drug exposure throughout therapy. At each occasion, a typical PK profile was obtained, including three plasma samples collected approximately pre-dose (trough or C_min_), 30 min and 5 to 6 h after daptomycin administration. On some occasions, only one or two samples could be obtained. Sampling times were recorded precisely in each patient. Daptomycin plasma concentrations were measured using an HPLC assay with a photodiode array detector. The method was adapted from a previous publication [21]. The lower limit of quantification was 2 mg/L. The bias was less than 8%, and the interday precision was lower than 11%.

### 2.3. Building of Daptomycin Nonparametric Population PK Model for BestDose

In our previous work, the final population PK model of daptomycin was built by using a parametric approach (SAEM algorithm in the Monolix software) [20]. The final model was a two-compartment model with body weight and P-gp genotype as covariates influencing the central volume of distribution, while daptomycin total body clearance was influenced by creatinine clearance and sex. We reanalyzed the original dataset in order to implement a nonparametric model into the BestDose software [22]. The NPAG algorithm embedded into the Pmetrics package for R was used to develop the nonparametric model [23,24]. The final nonparametric model was a two-compartment model parameterized with a central volume of distribution (V_1_, in L/70 kg), non-renal elimination rate constant (K_i_), renal elimination rate constant (K_s_, to be multiplied by creatinine clearance), and constants of rate transfer from the central to the peripheral compartment (K_cp_) and backward (K_pc_). Therefore, the final nonparametric model only included two covariates, body weight and creatinine clearance. The error model was based on the assay error pattern. The predictive performance of the final model was assessed by computing the bias and precision of model-based individual predictions (see below). The model was then imported into the Windows version of the BestDose software.

### 2.4. Implementation of A Parametric Model in Tucuxi

As we aimed at comparing our nonparametric model to a reference model for TDM and dose adjustment, we implemented the seminal daptomycin parametric model developed by daptomycin’s manufacturer [25] into the Tucuxi software. Tucuxi is a new software dedicated to the analysis of TDM results and individual dose adjustment (http://www.tucuxi.ch/, accessed 27 December 2021) [26].

It was developed by the School of management and engineering of Vaud (HEIG-VD) and the University Hospital of Lausanne (CHUV), Switzerland. Tucuxi only runs parametric models. It offers a collection of population PK models and also allows new models to be imported.

The two-compartment reference model from Dvorchik et al. [25] was imported into Tucuxi, including its error model and all its covariates except body temperature, which influenced daptomycin clearance in the original model. This covariate was discarded because it is not measured consistently in all patients in routine, especially in outpatients, and its value may be altered by co-treatment such as acetaminophen. The other covariates were retained, with creatinine clearance and sex influencing daptomycin clearance and body weight influencing both intercompartment clearance and peripheral volume of distribution.

### 2.5. External Validation and Comparison of the Two Models

We used an external dataset from 94 patients who received daptomycin for BJI and had TDM to validate the predictive performance of our nonparametric model in BestDose and that of the parametric model in Tucuxi. In this evaluation, we only used the daptomycin concentrations collected during the first TDM occasion in each patient.

The dosage history, covariate values, times and values of measured concentrations in each patient were entered into each program. The population model was fit to individual data by using Bayesian regression in both tools. After estimation of Bayesian posterior individual PK parameters, the estimated PK profile of each patient was obtained, including predictions of measured daptomycin concentrations.

Bias and precision of model-based individual predictions were assessed by computing the mean error (ME) and mean absolute percent error (MAPE) of prediction, respectively, as follows:

(1) $$\mathrm{ME}=\frac{\sum_{i=1}^{n}{\mathrm{Cpred}}_{i}-{\mathrm{Cobs}}_{i}}{n}$$

(2) $$\mathrm{MAPE}=\frac{\sum_{i=1}^{n}\left|\frac{{\mathrm{Cpred}}_{i}-{\;\mathrm{Cobs}}_{i}}{{\mathrm{Cobs}}_{i}}\right|}{n}$$

where Cpred_i_ are the concentrations predicted by the model, Cobs_i_ are the observed concentrations, and n is the total number of observations.

ME and MAPE were compared between BestDose and Tucuxi by using the Wilcoxon signed-rank test for paired samples. We also compared the regression of predicted versus observed concentrations from each model.

Because daptomycin AUC is an important predictor of its antibacterial effect, we also compared the estimations of individual daily AUC (AUC_24h_) in the 94 patients provided by BestDose and Tucuxi.

Finally, we compared the individual daily doses computed by the two programs for achieving two targets at the steady-state in each patient: the minimum effective dose (D_min_) necessary to attain AUC_24h_ = 666 mg.h/L and the maximum effective dose (D_max_) associated with C_min_ = 24.3 mg/L. The AUC target was associated with the bactericidal effect of daptomycin in an animal model of staphylococcal infection [15], while the C_min_ target was the cut-off value associated with increased risk of CPK elevation in humans [14].

The Mann–Whitney test was used for comparisons of AUC_24h_, half-life, central volume of distribution of daptomycin, as well as predicted doses between the two programs.

In addition, we assessed the concordance between predicted concentrations, AUC_24h_, D_min_ and D_max_ by performing a Bland–Altman analysis [27]. For each pair of predicted concentrations, AUC_24h_, D_min_ and D_max_ provided by BestDose and Tucuxi, we plotted the difference (BestDose estimate minus Tucuxi estimate) versus the average value. We used the BA-plotteR webtool to build the scatterplots and derive the mean difference line and limits of agreement [28]. Regression-based limits of agreement were used for the plot of AUC_24h_ and Dmax because the slope of the regression line was significantly different from zero, suggesting a proportional bias.

## 3. Results

### 3.1. Population Data

A total of 841 plasma concentrations of daptomycin collected in 175 patients were available for analysis. The learning and validation datasets included 577 observations from 81 patients and 264 observations from 94 patients, respectively. The characteristics of the two populations are summarized in Table 1.

In the validation dataset, the mean daptomycin dosage was 7.6 ± 1.3 mg/kg per day, which is consistent with guidelines. The mean AUC_24h_ estimated with BestDose was 975 ± 395 mg.h/L on the first TDM occasion, which is higher than the efficacy threshold (AUC_24h_ ≥ 666 mg.h/L), for a putative MIC of 1 mg/L. However, 21.3% of patients were underexposed (AUC_24h_ < 666 mg.h/L). On the other hand, 31.9% of patients had C_min_ > 24.3 mg/L and may be considered as overexposed.

### 3.2. Population Modeling in the Learning Dataset

**Figure 1:**
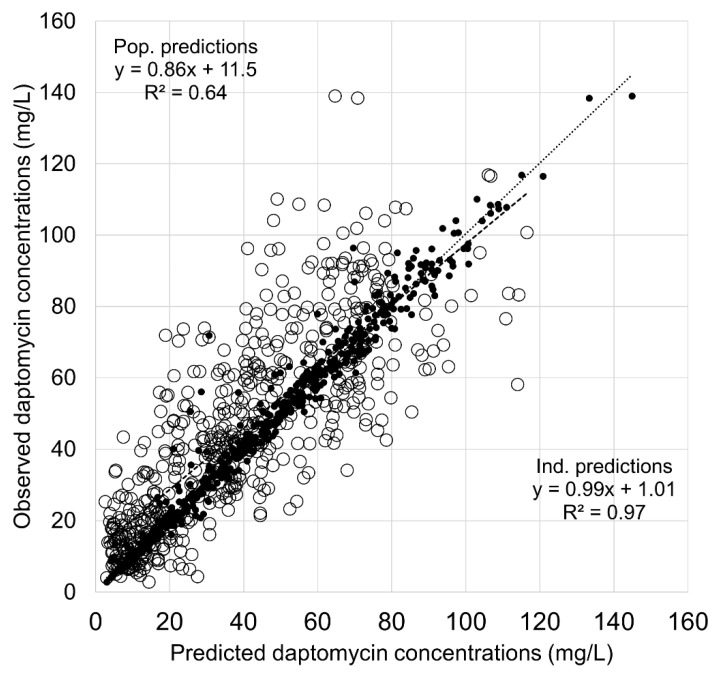
Observed versus model-based predicted concentrations of daptomycin in the learning dataset. Open circles and dashed lines, population predictions; black dots and dotted lines, individual predictions. Abbreviations: Ind., individual; Pop., population.

The parameter values of the nonparametric population model built with Pmetrics in the learning dataset are shown in Table 2. Of note, as the real parameter distributions are discrete, the means and standard deviations of parameters cannot provide a complete description of the distributions. The final nonparametric model described the data very well in the learning dataset with ME of −0.72 ± 4.3 mg/L and MAPE of 6.3 ± 8.1%. The plot of observed concentrations versus population and individual predictions is shown in Figure 1.

### 3.3. Comparison of BestDose and Tucuxi Models in the Validation Dataset

**Figure 2:**
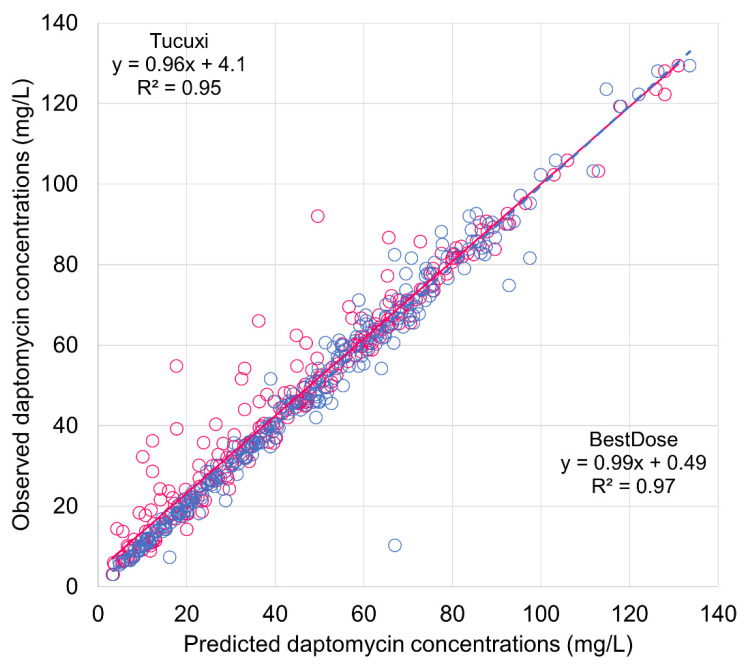
Observed plasma concentrations of daptomycin versus individual predictions from BestDose and Tucuxi in the validation dataset. Blue circles and dashed lines, BestDose predictions; red circles and solid line, Tucuxi predictions.

The model predictions computed with BestDose in the validation dataset were as good as in the learning set, with ME = −0.087 ± 5.1 mg/L and MAPE = 7.8 ± 34.7%. The parametric model implemented in Tucuxi also described the validation data very well with ME = −2.3 ± 6.0 mg/L and MAPE = 9.9 ± 13.3%. The ME and MAPE were slightly higher than that from the BestDose model, and the differences were significant (*p* < 0.001 and *p* = 0.0022 for ME and MAPE, respectively). Figure 2 shows the plot of observations versus individual predictions from both models, which look very similar.

The Bayesian estimations of daptomycin concentrations, AUC_24h_, D_min_, D_max_, as well as the estimates of the main PK parameters (V_1_, half-life and total body clearance) calculated with both programs are summarized in Table 3. Regarding PK parameters estimates, BestDose provided higher mean estimates of V_1_ and half-life, while the mean clearances were similar. The correlation between estimates from the two programs was low.

**Figure 3:**
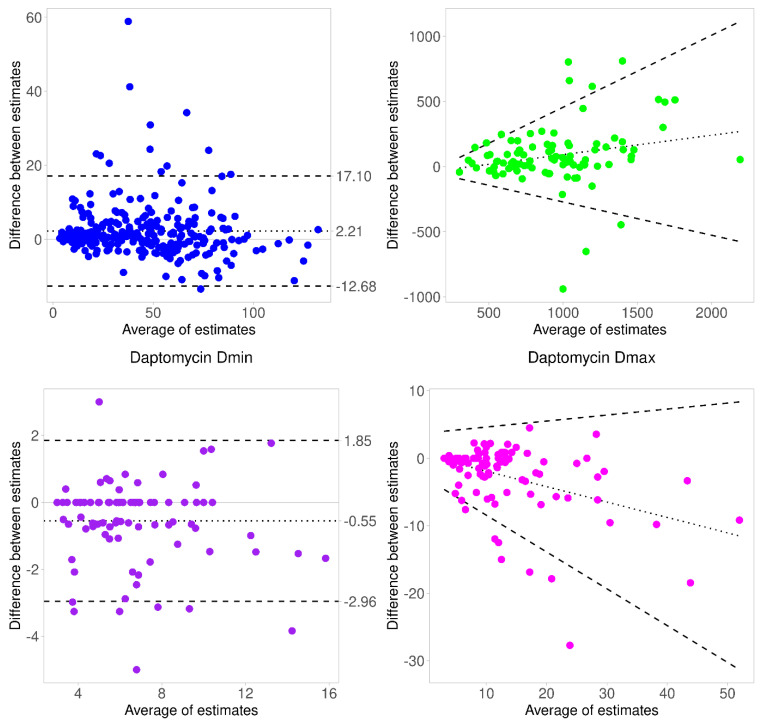
Bland–Altman plot assessing the agreement between estimates of daptomycin concentrations, AUC_24h_, D_min_ and D_max_ from BestDose and Tucuxi. The difference was calculated as BestDose estimate minus Tucuxi estimate in all subplots. Units of daptomycin concentrations, AUC_24h_, D_min_ and D_max_ are mg/L, mg.h/L, mg/kg and mg/kg, respectively. The dotted line is the regression line; the dashed lines are the limits of agreement. Regression-based limits of agreement were used for plots of daptomycin AUC_24h_ and Dmin because the slope of the regression was significantly different from zero, suggesting proportional bias.

Predicted daptomycin concentrations from BestDose correlated well with those from Tucuxi and showed a good agreement, as displayed in the Bland–Altman plot (Figure 3). A few values were above the upper limits of agreement, those corresponding to underestimation of observed concentrations by the Tucuxi model, as one can observe in Figure 2. The AUC_24h_ estimated by BestDose were not significantly different than those from Tucuxi. While the linear correlation between AUC_24h_ estimates was moderate, the Bland–Altman analysis showed a good agreement. The minimum effective doses (D_min_) required to achieve the target AUC of 666 mg.h/L were not significantly different, and the correlation and agreement were acceptable. By contrast, the maximum effective doses (D_max_) associated with C_min_ = 24.3 mg/L were substantially different (*p* = 0.046) between the two programs, with higher doses suggested by Tucuxi on average. The Bland–Altman plot (Figure 3) confirmed a trend to an increasing difference between Tucuxi and BestDose D_max_ estimates for ascending doses.

## 4. Discussion

Daptomycin is increasingly used as an alternative to vancomycin for treating systemic infections caused by Gram-positive bacteria, including BJI [29]. Considering the established exposure–response effect described for both antimicrobial effect and muscular toxicity, TDM of daptomycin appears to be a relevant approach for evaluating and optimizing drug exposure [30]. In addition, there is a need for pharmacometric tools to analyze TDM results and perform precision dosing of this drug. In this study, we implemented and compared two Bayesian dosing programs for daptomycin TDM based on data from patients with BJI.

First, our analysis confirmed the interest in daptomycin TDM and dosage individualization, as the achievement of exposure targets was not optimal. In the validation group, 21.3% of patients did not achieve the AUC_24h_ target on the first TDM occasion after initial dosing, and 31.9% had C_min_ values above the safety target. Urakami et al. reported a low rate of efficacy target attainment (40%) in patients who received 6 mg/kg [31]. In another prospective TDM study in patients who received daptomycin for bacteremia, 9.5% of individuals showed C_min_ > 24.3 mg/L. Half of those presented an elevation of CPK [30]. Those published results and ours confirm that exposure to daptomycin is often suboptimal and that dosage individualization based on TDM is desirable.

We developed a nonparametric PK model of daptomycin for the BestDose software. Compared with the original model previously published by our group, the model implemented with BestDose was simpler, with only two covariates. Despite this simplification, the model performance was very good, with low bias and imprecision in individual predictions of daptomycin concentrations in both the learning and validation datasets. This illustrates the relative importance of concentration measurements and covariates in the Bayesian estimation of PK parameters. As shown by Sheiner and colleagues, measured drug concentrations are much more informative about PK parameters than covariates in the Bayesian estimation [32]. Therefore, models to be used for TDM and individual Bayesian estimation do not necessarily require many covariates. By contrast, covariates are more important for population predictions and model-based initial dosing.

Our nonparametric model implemented into BestDose compared well with the reference model from Dvorchik et al. implemented in Tucuxi [25]. Indeed, the BestDose model performed slightly better than the Tucuxi model in estimating daptomycin concentration in the validation group. This may be due to the population used in model building, as our model was built in a similar population of patients with BJI, while the model from Dvorchik et al. was based on data from 15 clinical studies conducted in healthy volunteers and patients without BJI. In addition, the assay method was the same in the learning and validation dataset for the model implemented in BestDose, which was not the case for the model in Tucuxi. The predicted concentrations, D_min_ and estimated AUC_24h_, were similar between the two programs and showed a good agreement. The dose suggested is probably the most relevant parameter to be compared between two dosing programs, as it directly influences dosing decisions and patient care. The doses calculated by the two programs for achieving the target AUC_24h_ (D_min_) were remarkably consistent. By contrast, the maximal effective doses associated with C_min_ still below the safety target were somewhat different, with higher D_max_ values predicted by Tucuxi. This difference may be explained by differences in model parameterization, algorithm and statistical distribution between the two programs. As shown in Table 3, the BestDose model tended to estimate longer half-life values than the Tucuxi model. As a result, the BestDose model would predict a higher plasma trough concentration for a given dosage than the Tucuxi model, all things being equal. We believe that this difference is unlikely to be an issue for clinical use of the two programs, as targeting such a high C_min_ would not be the primary goal. Users are expected to target the minimum effective dose (D_min_) necessary to achieve the AUC efficacy target and could adjust the dosing interval in order to maintain C_min_ below the safety target if necessary.

Factors other than the original population may also explain the differences in estimates and parameter values from the two models. The BestDose model was parameterized with rate constants, as required by the program, while the model implemented in Tucuxi was parameterized with CL, in agreement with the original publication. Moreover, the statistical nature of the two models is different, as the model implemented in BestDose is nonparametric, while the model imported into Tucuxi is parametric. This statistical feature influences the Bayesian estimation of individual PK parameters and the computation of the dosage for a given target. With a nonparametric model, the entire discrete Bayesian posterior distribution is used in model fitting and dosage calculation [24,33]. With a parametric model, only a single value of the continuous Bayesian posterior distribution is used in such calculation, which is the maximum a posteriori (MAP) [34]. Despite those technical differences, the two models provided overall comparable performance in the estimation of daptomycin concentrations and AUC_24h_ and in the calculation of the dose required to achieve the efficacy target.

This study has several limitations. Data were collected during routine patient care, so errors may have occurred in the collection. The population only included patients with BJI, so the nonparametric model developed for BestDose may not perform as well in other patients’ groups. Based on the relatively good performance of the model implemented in Tucuxi in a population different from the original, one can expect similar robustness of our model, but further research is necessary to confirm this. It is likely that specific models would be required for daptomycin dosing in unstable patients such as critically ill patients. Moreover, the estimation of individual PK parameters, AUC values and doses depends on the sampling design, which was quite consistent in our patient population, with three concentrations measured in most patients at similar time-points. The results might be different with another sampling design. Finally, we considered *Staphylococcus aureus* as the targeted pathogen, and the target AUC was based on this assumption. A lower AUC/MIC ratio was suggested as a daptomycin PK/PD target for enterococcal bacteremia [35]. In such a case, the effective dose suggested by the PK models would have been different.

## 5. Conclusions

In conclusion, our results suggest that daptomycin dosing can be optimized in patients with BJI, as significant proportions of underexposure and overexposure were observed after initial dosing based on patients’ weight. Two pharmacometric tools for model-based TDM and dosage adjustment of daptomycin were developed and externally validated in patients with BJI. The two models showed good predictive performance and overall comparable results. They are available in the BestDose and Tucuxi programs for precision dosing of daptomycin.

## Figures and Tables

**Table 1:** Population characteristics.

Variables	Learning Dataset (*n* = 81)	Validation Dataset (*n* = 94)
Proportion of women/men	41.9%/58.1%	42.5%/57.5%
Age (years)	60 ± 18	62 ± 17
Body weight (kg) ^a^	79 ± 20	76 ± 18
CL_CR_ (mL/min) ^a, b^	100 ± 41	103 ± 56
Initial dose of daptomycin (mg/kg/24 h)	8.0 ± 1.9	7.6 ± 1.3
Number of TDM occasions per patient	2.5 ± 7.9	2.8 ± 0.5
AUC_24h_ (mg.h/L)	ND	975 ± 395
AUC_24h_ < 666 mg.h/L (%)	ND	21.3%
C_min_ (mg/L)	ND	21.3 ± 12.9
C_min_ > 24.3 mg/L (%)	ND	31.9%

Data are given as mean ± standard deviation unless otherwise stated. ^a^ Values of body weight and creatinine clearance are those collected on the first TDM occasion. ^b^ CL_CR_ is creatinine clearance estimated by the Cockcroft–Gault equation. Abbreviations: ND, not determined; TDM, Therapeutic drug monitoring

**Table 2:** Population parameter values of daptomycin in the learning dataset (Pmetrics estimation).

Parameter ^a^	Mean	Median	Variance	Coefficient of Variation
V_1_ (L per 70 kg)	6.90	7.18	7.40	39.4%
K_s_ (h^−1^ per 100 mL/min of CL_CR_)	0.050	0.045	0.0020	89.6%
K_i_ (h^−1^)	0.060	0.052	0.0025	83.2%
K_cp_ (h^−1^)	0.693	0.287	0.669	118.0%
K_pc_ (h^−1^)	0.667	0.449	0.424	97.7%

^a^ V_1_ is daptomycin central volume of distribution, K_s_ and K_i_ are the renal and non-renal components of the elimination rate constant, K_cp_ and K_pc_ are the intercompartment transfer rate constants. The elimination rate constant of daptomycin (K_e_) was described as follows: K_e_ = K_s_ ∗ (CL_CR_/100) + K_i_.

**Table 3:** Comparison of individual PK estimates and parameters from BestDose and Tucuxi.

PK Quantity	BestDose Estimate	Tucuxi Estimate	*p*-Value	Determination Coefficient (R^2^) ^a^
Predicted concentrations (mg/L)	46.6 ± 46.7	44.3 ± 44.5	0.29	0.93
AUC_24h_ (mg.h/L)	975 ± 395	893 ± 345	0.19	0.66
D_min_ (mg/kg)	6.4 ± 2.7	6.9 ± 2.8	0.14	0.81
D_max_ (mg/kg)	12.0 ± 8.6	14.7 ± 10.7	0.046	0.77
V_1_ (L)	7.9 ± 3.5	5.9 ± 2.4	<0.001	0.20
T_1/2_ (h) ^b^	16.8 ± 10.6	13.1 ± 4.7	0.015	0.11
CL_dap_ (L/h) ^c^	0.74 ± 0.42	0.73 ± 0.29	0.50	0.31

Data are given as mean ± SD, unless otherwise stated. ^a^ Linear correlation between BestDose and Tucuxi estimates. ^b^ Terminal half-life. ^c^ Daptomycin total body clearance. Of note, CL_dap_ was not directly estimated by BestDose but calculated based on estimates of V_1_ and elimination rate constant.

## Data Availability

Data are available from the authors upon request.
